# Study of the distribution of *Glycyrrhiza uralensis* production areas as well as the factors affecting yield and quality

**DOI:** 10.1038/s41598-023-31946-5

**Published:** 2023-03-29

**Authors:** Xinping Cui, Lin Lou, Yan Zhang, Binbin Yan

**Affiliations:** 1 State Key Laboratory and Breeding Base of Dao-di Herbs, Resource Center of Chinese Materia Medica China Academy of Chinese Medical Sciences, Beijing, 100700 China; 2 School of Chinese Pharmacy, Beijing University of Chinese Medicine, Beijing, 100102 China

**Keywords:** Plant sciences, Ecology

## Abstract

Wild licorice in China is mainly distributed in northern China, such as Gansu, Ningxia, and Inner Mongolia Provinces. The origin of wild licorice has varied among historical periods. The cultivated origin of planted licorice has the same as 59.26% of wild licorice. The distribution of cultivated licorice was shifted to the northwest relative to that of wild licorice. The yield and quality of cultivated licorice vary greatly from different origins, showing a certain pattern of variation from west to east. The same batch of licorice seedlings was planted at 8 sites overlapping the main licorice production areas in China. The yield and quality of licorice in the Baicheng experimental plot were low. The yield of licorice in the Jingtai and Altay experimental plots was high, but the quality was poor. The quality of licorice in Chifeng and Yuzhong experimental sites was high, but the yield was low. Principal component analysis of environmental and soil factors generated five characteristic roots with a cumulative contribution rate of 80%, three of which were related to soil and referred to as the soil charge factor, soil water factor, and soil nutrient factor, and the load coefficients of the water and nutrient factor were the largest. Soil conditions, especially water and nutrients, might have a substantial effect on the observed changes in the licorice production area. Generally, the regulation of water and nutrients merits special attention when selecting areas for the production and cultivation of licorice. This study can provide reference for the selection of cultivated licorice production areas and the research of high-quality cultivation techniques.

## Introduction

Gancao medicinal material comprises the dry roots and rhizomes of *Glycyrrhiza uralensis* Fisch.*, Glycyrrhiza inflata* Batalin. *or Glycyrrhiza glabra* L. It is a commonly used ancient Chinese medicine and a bulk export commodity. It is also widely used as an ingredient in food, cosmetics, and other products^1–4^. It has long been known as the "king of medicine" and "national elder"^5^. *G. uralensis* Fisch. is the main species that have been introduced and cultivated in China^6^. Description of the characters of *G. uralensis* Fisch. in ancient books and comparison with the shapes of the three Glycyrrhiza plants indicate that the variety used historically is *G. uralensis* Fisch.^7^. Licorice occurs in 13 provinces and autonomous regions, primarily in the three northern regions, and its distribution spans 10 degrees of latitude and 52 degrees of longitude^8^. There is substantial variation in climate, soil, and other eco-environmental conditions among regions^8^, and this variation can result in low and unstable yields; the content of active ingredients in cultivated licorice is affected by the ecological environment, and substantial differences have been observed among production areas^9–12^. Cultivated licorice often does not meet the quality standards specified in the Chinese Pharmacopoeia^13^. No studies to date have characterized historical changes in the distribution of wild licorice. Comparison of the distribution of wild licorice with the main production area of cultivated licorice can clarify the effect of climate and soil conditions on licorice yield and thus aid the selection of appropriate sites for licorice cultivation.

Suitable environmental conditions can affect the yield and quality of medicinal materials. Climate and soil factors have a substantial effect on the growth of medicinal plants and the accumulation of primary metabolites^14,15^, and temperature and precipitation are particularly important climatic variables. For example, the main climate factors affecting the growth and development of licorice in the Hexi Corridor and Inner Mongolia are heat and rainfall^16^. The annual average temperature and the maximum temperature in July are negatively correlated with the content of glycyrrhizic acid, glycyrrhizin, and isoglycyrrhizin, and the altitude and average annual precipitation is positively correlated with this three effective components^17^. Although licorice can grow naturally in areas with precipitation less than 100 mm to 500 mm, the optimal annual precipitation^18^ is 100–300 mm, the optimal average annual growth temperature is 4–8 °C, and the annual accumulated temperature of ≥ 10 °C is 3000–3800 °C, and these are consistent with the climatic conditions of Gansu, Ningxia, and Inner Mongolia Provinces^19^. The soil water content is an important factor affecting the growth of licorice. Within a certain range, the content of glycyrrhizin in licorice is positively correlated with soil water^20,21^. Drought stress reduces plant photosynthesis and affects plant growth^22–24^. In potted licorice plants, the content of glycyrrhizic acid is lowest when the soil moisture is 12% and decreases when it is higher than 14%^25^. The contribution of soil basic fertility to the formation of licorice yield is not different from that of basal fertilizer applied to the soil before transplanting, indicating that yield increases with fertilizer input. Li et al.^26^ found that the effect of soil basic fertility on licorice yield is 47.8% in Yanchi new irrigation areas, and the dependence on basal fertilizer before transplanting is 52.2%.

Several studies have shown that the quality of licorice varies among production areas. To explore the effects of climate and soil factors on the yield and quality of licorice, variation in the historical distribution of wild licorice was characterized, and the yield and quality of licorice in the main areas of licorice cultivation in different regions of the country were compared. One-year-old licorice seedlings from the same source were used for multi-point field experiments in the main production areas of licorice in China, and differences in the underground growth indexes and content of active ingredients of licorice under different environments were determined. The climate and soil factors that mainly affect the yield and content of active ingredients of licorice were identified. The results of this study will aid the selection of sites for licorice cultivation as well as research and development of high-quality cultivation techniques.

## Results

### Distributional changes in licorice production areas

**Figure 1:**
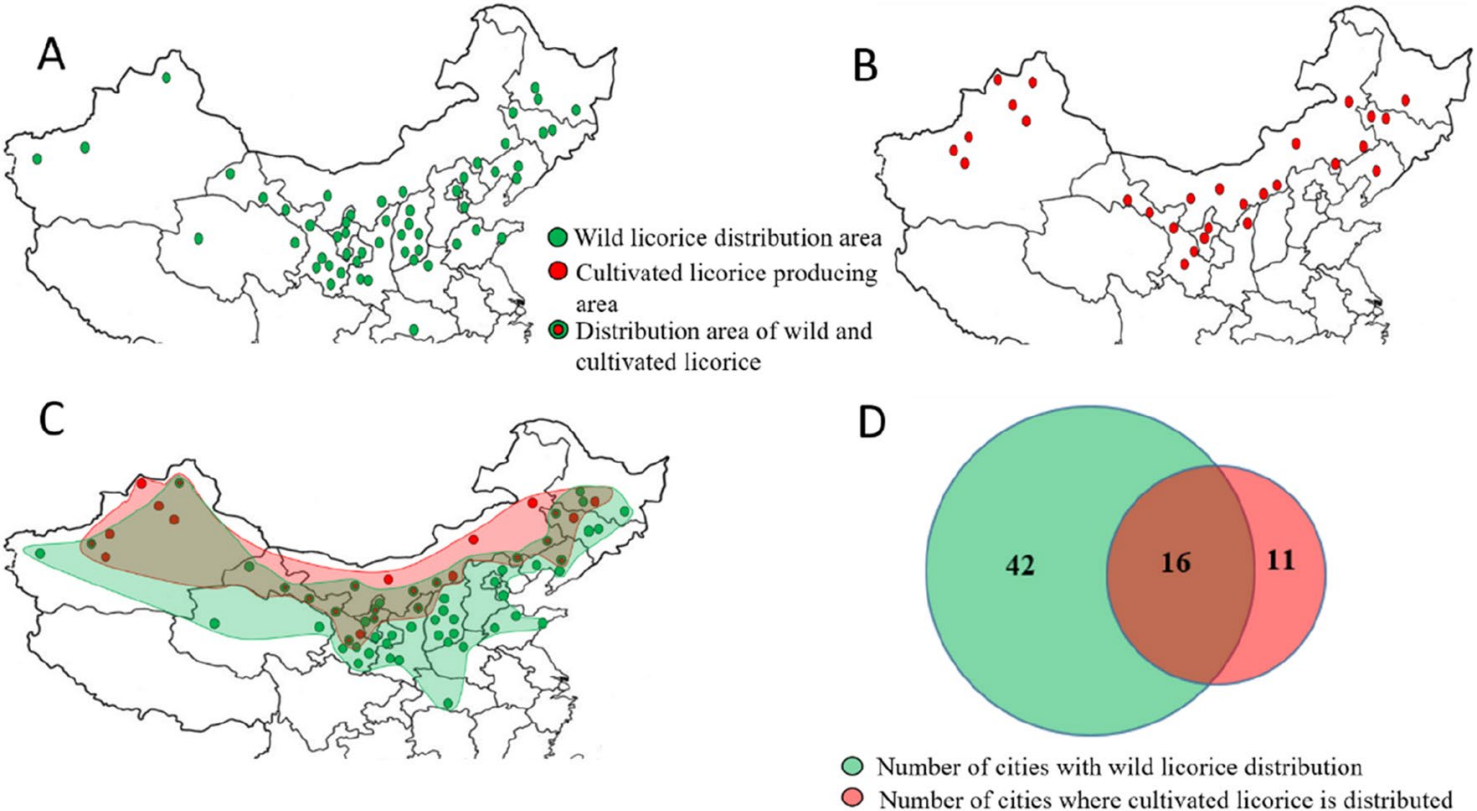
The distribution of wild licorice and cultivated licorice in China. (**A**) The distribution of wild licorice, (**B**) The distribution of cultivated licorice, (**C**) The difference between the distribution of wild licorice and cultivated licorice. The green area in the figure represents the distribution of wild licorice, and the pink area represents the distribution of cultivated licorice. (**D**) The number of cities where wild and cultivated licorice has been. (**A**–**C**) is a map of China, but it does not include all administrative regions of China.l.

In China, wild licorice is mainly distributed in northern areas such as Gansu, Ningxia, and Inner Mongolia Provinces. According to ancient books, Jingzhou City, Hubei Province is the southernmost city where wild licorice occurs (Fig. 1A and Table S1). Cultivated licorice is also distributed in northern China (Fig. 1B). The distribution of cultivated licorice is shifted to northwestern China relative to that of wild licorice (Fig. 1C). Wild licorice has been recorded in a total of 58 cities. The distribution of wild licorice has varied among the different dynasties (Table S1); it was the smallest in the Han Dynasty (2 seats) and the largest in the Republic of China (31 seats). Excluding the influence of human factors such as ancient war, there has been a gradual increase in the size of the distribution (Table 1). Cultivated licorice has been recorded in 27 cities, wild licorice has been recorded in 42 cities, and both wild and cultivated licorice have been recorded in 16 cities; 59.26% of the cities have cultivated licorice, and 72.41% of the cities have wild licorice (Fig. 1D). This indicates that the growth environment of wild licorice can be used to select licorice cultivation areas.

**Table 1:** Number of cities with licorice during different periods.

Period	Number of cities/seats
Han dynasty	2
Tang dynasty	10
Song dynasty	15
Yuan dynasty	3
Ming dynasty	8
Qing dynasty	25
From the Republic of China to the present	31

### Analysis of the yield and quality of cultivated licorice in China

**Figure 2:**
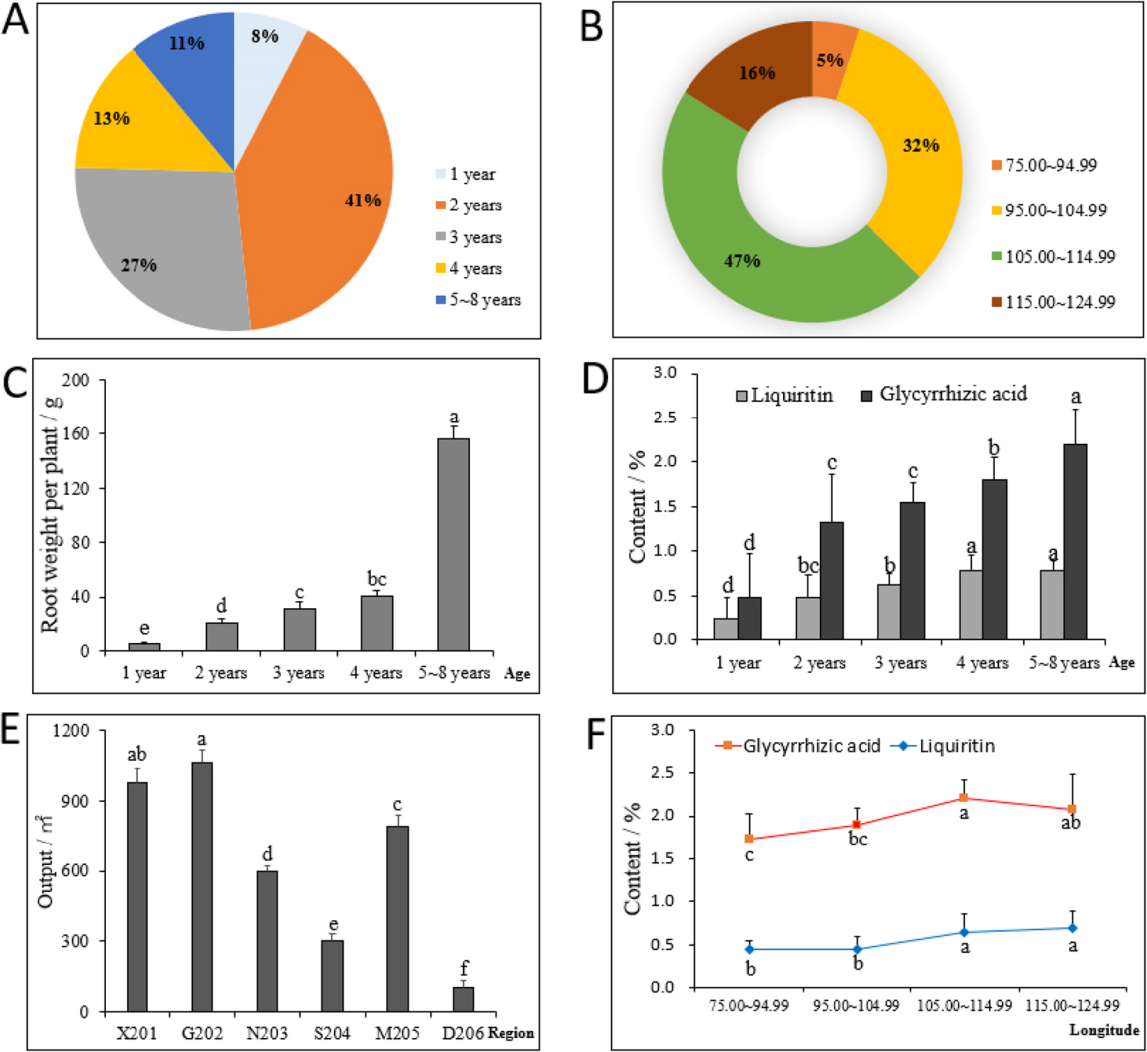
Analysis of the yield and quality of cultivated licorice in China. (**A**) The age distribution of licorice samples collected in the study, (**B**) The longitudinal distribution of licorice samples, (**C**) The root weight of licorice at different ages, (**D**) The content of glycyrrhizic acid and glycyrrhizin in licorice at different ages. (**E**) The output of licorice in different provinces (in the sample number, the first letter X refers to “Xinjiang,” G refers to “Gansu,” N refers to “Ningxia,” S refers to “Shaanxi,” M refers to “Inner Mongolia Autonomous Region,” and D refers to the three provinces in Northeast China. The first digit indicates that the age of the collected sample was 2 years; the values from the second to the third digit indicate the sample number under the first letter), (**F**) Refers to the content of glycyrrhizic acid and glycyrrhizin in licorice in different longitudinal ranges. Different letters in the figure indicates significant differences between groups.

The ages of licorice samples collected in this survey ranged from 1 to 8 years, and samples from 2-year-old plants were the most common, accounting for 41% of all samples, followed by samples from 3-year-old and 1-year-old plants (Fig. 2A). The samples were mainly distributed in the longitudinal range of 75.00–124.99, especially 105.00–114.99, which included 47% of the samples (Fig. 2B). As the growth age increased, the single plant weight of licorice and the content of glycyrrhizic acid and glycyrrhizin gradually increased (Fig. 2C and D). The first repetition, the second repetition, and the third repetition of the 2-year-old licorice yield in the same provinces were pooled, and their average values were compared with those shown in Fig. 2E. The yield of licorice from different production areas varied greatly and decreased from west to east. Calculation of the average value of the glycyrrhizin and glycyrrhizic acid content of 2-year-old licorice samples within the fixed longitudinal range revealed that the content of these compounds increased gradually with longitude (Fig. 2F). In general, the yield and quality of cultivated *G. uralensis* varied among production areas and increased with longitude. Because of variation in the provenance of *G. uralensis* and field management technology, the main factors contributing to this pattern cannot be inferred.

### Differences in the growth and yield of G. uralensis from the same provenance and different producing areas

**Figure 3:**
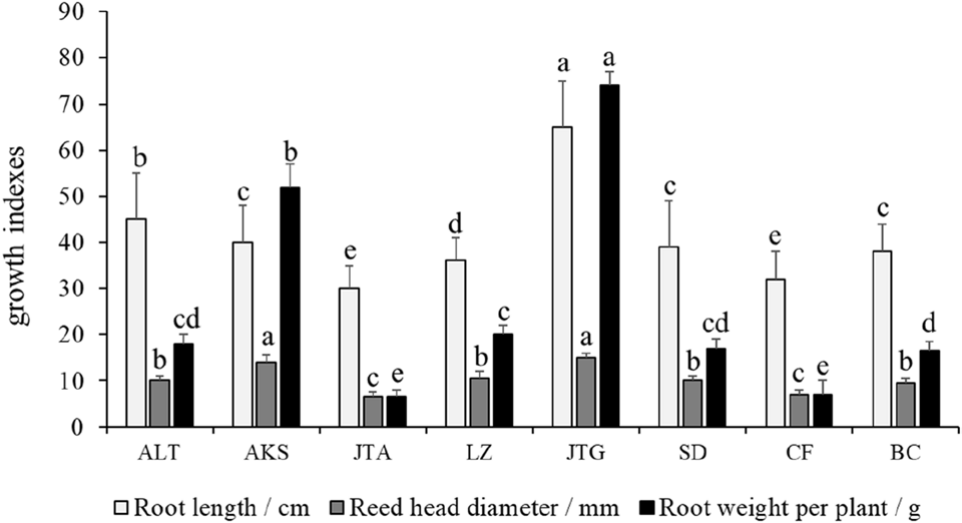
Differences in the growth and yield of licorice from different production areas. Different letters in the figure indicate significant differences between columns.

**Figure 4:**
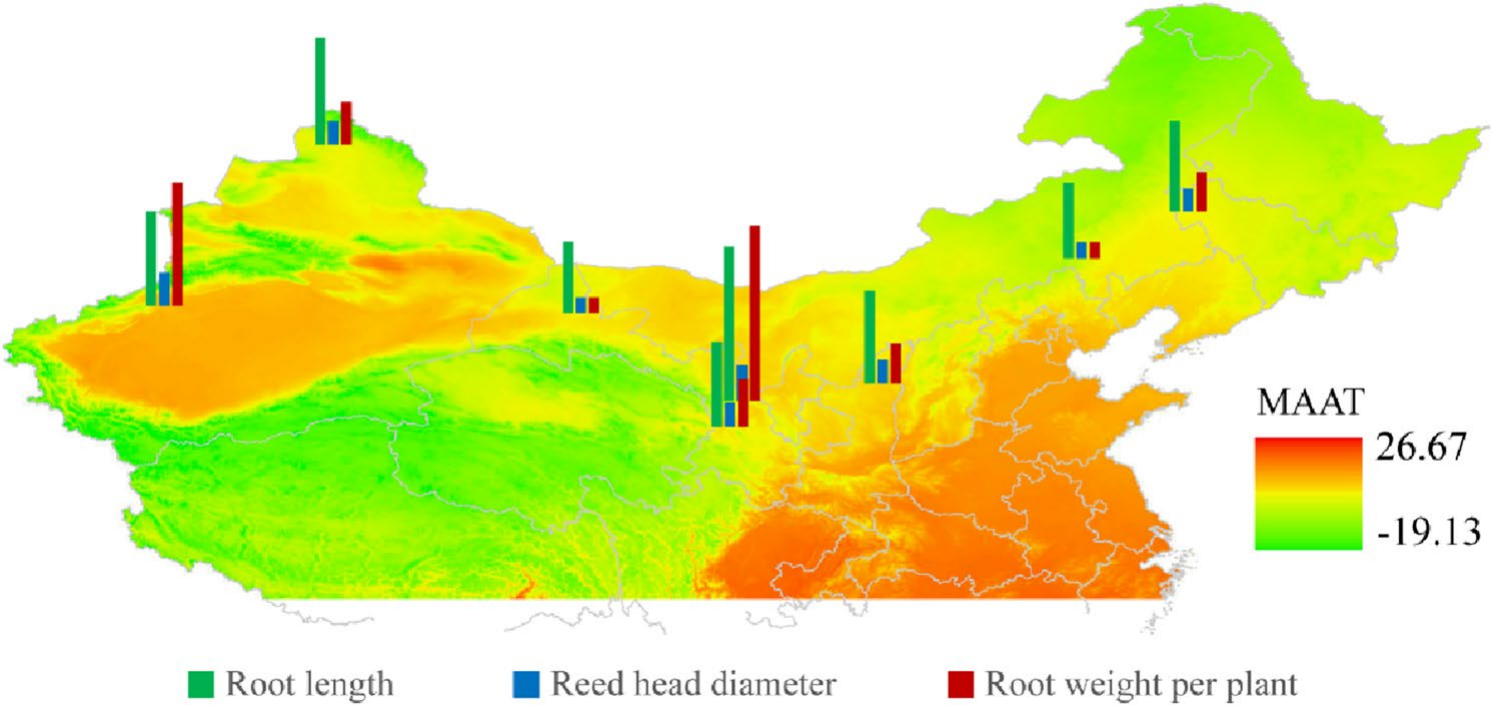
Schematic diagram of licorice growth and yield in different production areas based on Arc GIS. Coloring the map based on mean annual temperature data (MAAT) (Arc GIS 10.8--https://enterprise.arcgis.com/zh-cn/server/10.5/install/linux/about-connecting-to-arcgis-server-in-arcgis-for-desktop.htm).

A comparative analysis of growth and yield indexes of biennial licorice from the same provenance and different production areas are shown in Fig. 3. In addition, as shown in Fig. 4, the growth status of glycyrrhiza uralensis from different regions is spatially illustrated based on Arc GIS. There were significant differences in root length, reed head diameter, and root weight per plant of licorice from different production areas (*P* < 0.05). The root length, reed head diameter, and single plant root weight of *G. uralensis* at Jingtai experimental site (JTG) were the largest, followed by Altay experimental site (ALT), Jinta (JTA), Chifeng (CF), and Baicheng area (BC).

### Differences in the quality indexes of cultivated licorice from the same provenance and different producing areas

**Figure5:**
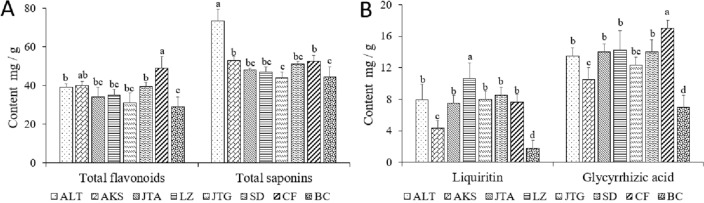
Differences in the quality indexes of cultivated licorice from the same provenance and different producing areas. (**A**) Content of total flavonoids and total saponins in different environments. (**B**) Content of glycyrrhizin and glycyrrhizic acid in licorice in different environments. Different letters in the figure indicate significant differences between columns.

**Figure 6:**
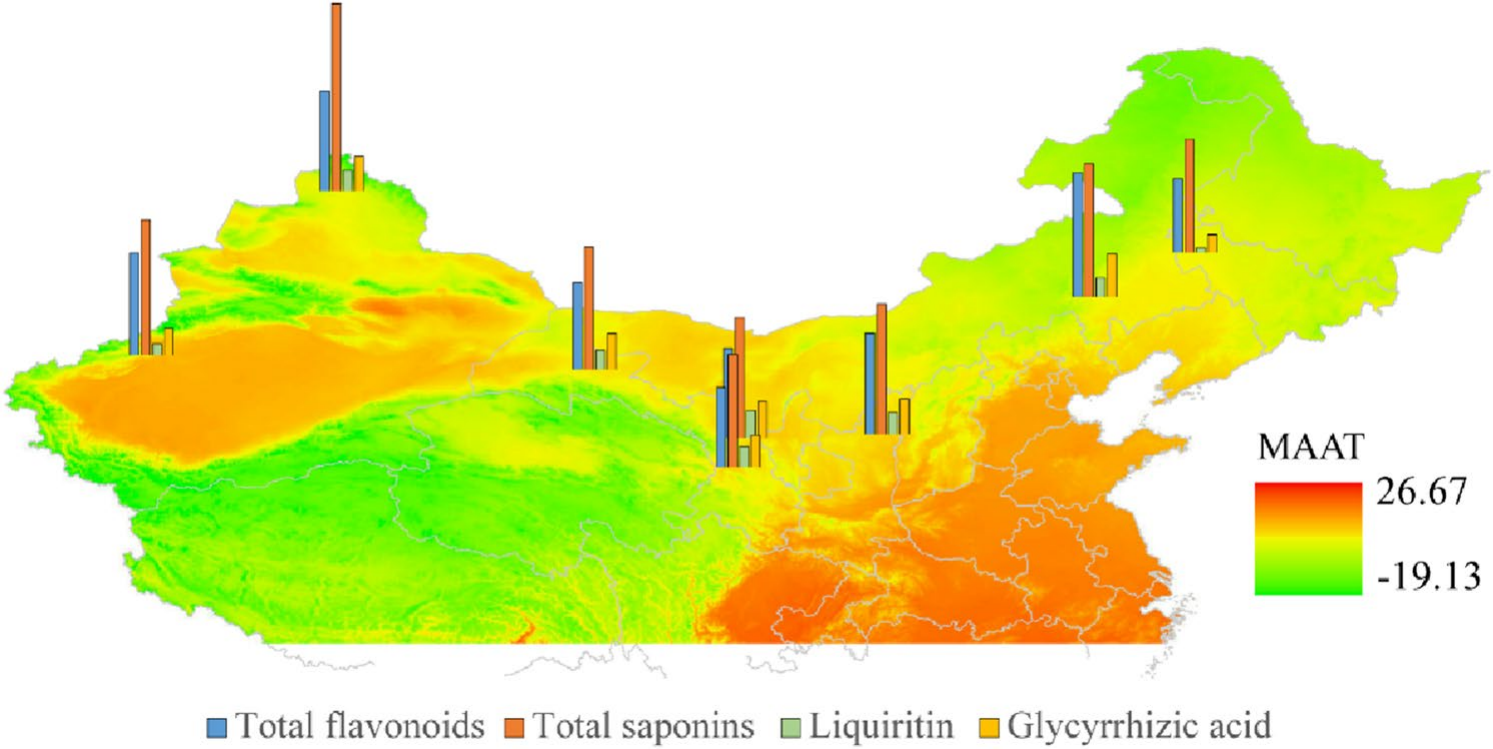
Schematic diagram of quality indicators of cultivated licorice from different origins of the same source based on Arc GIS. Coloring the map based on mean annual temperature data (MAAT) (Arc GIS 10.8--https://enterprise.arcgis.com/zh-cn/server/10.5/install/linux/about-connecting-to-arcgis-server-in-arcgis-for-desktop.htm).

Figure 5A shows differences in the content of total flavonoids and total saponins of biennial licorice from the same provenance and different production areas. There were significant differences in the content of total flavonoids and total saponins in licorice from different production areas (*P* < 0.05). The total flavonoids content of *G. uralensis* in Chifeng (CF) and Aksu (aks) was the highest, and that in Baicheng (BC) was the lowest. The total saponins content was highest in Altay (ALT); the lowest was in Jingtai (JTG) and Baicheng (BC), and there were no significant differences were observed in other regions. Figure 5B reveals significant differences in the content of glycyrrhizin and glycyrrhizic acid in licorice from different production areas (*P* < 0.05). The content of glycyrrhizin was highest in Lanzhou Yuzhong (LZ), the content of glycyrrhizic acid was highest in Chifeng (CF), and the content of glycyrrhizin and glycyrrhizic acid was lowest in Aksu (aks) and Baicheng (BC). Similarly, in Fig. 6, based on Arc GIS, the content of quality components of licorice from different areas is spatially illustrated.

### Comparative analysis of the HPLC fingerprints of cultivated G. uralensis from the same provenance and different producing areas

**Figure 7:**
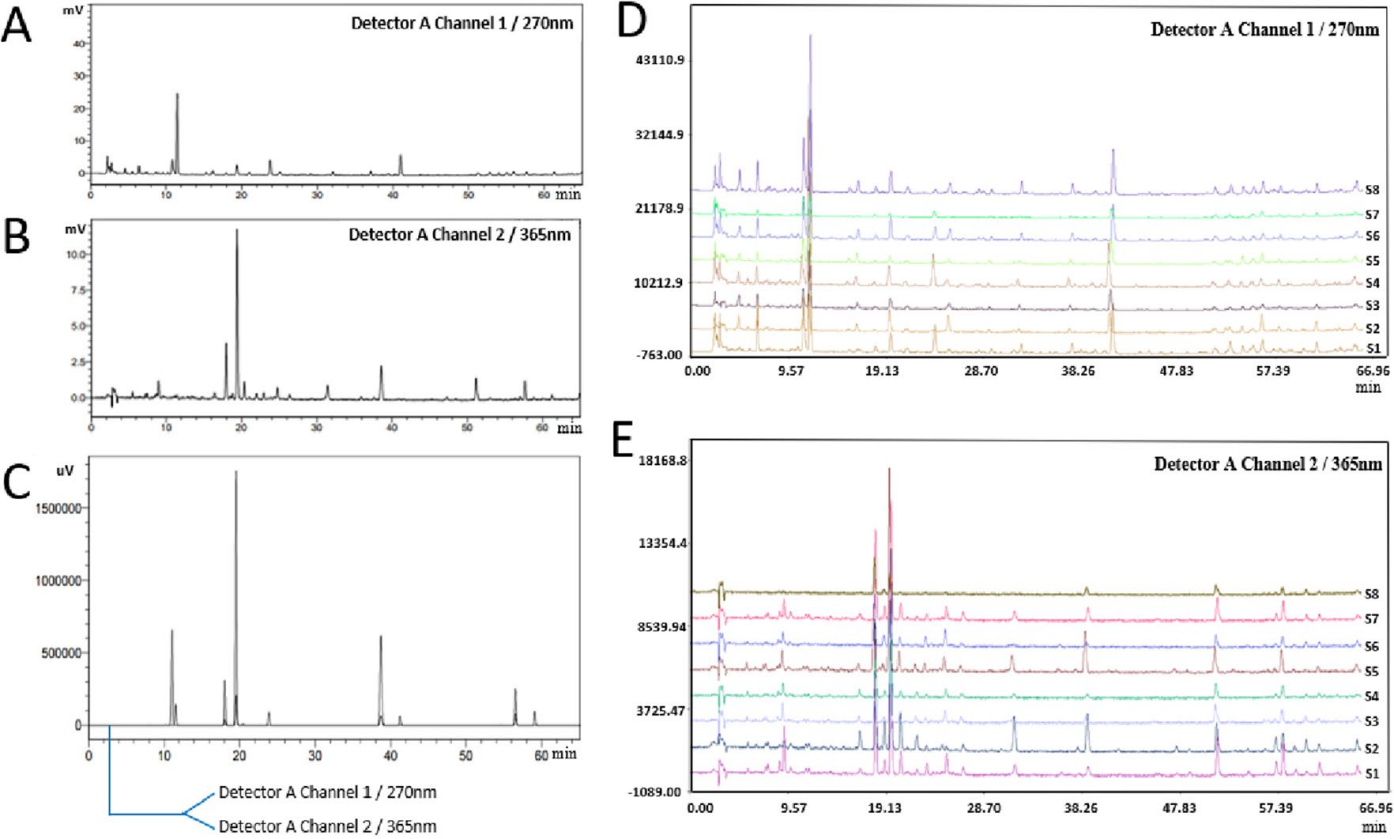
The HPLC fingerprints of cultivated G. uralensis from the same provenance and different producing areas. (**A**)The HPLC map of licorice at a wavelength of 270 nm; (**B**) The HPLC map of licorice at a wavelength of 365 nm; (**C**) The HPLC spectrum of the mixed standard; (**D**) The HPLC fingerprint of licorice from different production areas (270 nm); (**E**) The HPLC fingerprint of licorice (365 m).

Figure 7A and B show the HPLC spectra of licorice at the wavelengths of 270 nm and 365 nm, respectively. A comparison of the spectra of the mixed standard (Fig. 7C) confirmed that the peak with a retention time of 11.03 min was apigenin, the peak with a retention time of 11.55 min was glycyrrhizin, the peak with a retention time of 18.03 min was apigenin isoglycyrrhizin, the peak with a retention time of 19.51 min was isoglycyrrhizin, the peak with a retention time of 23.84 min was glycyrrhizin, the peak with a retention time of 38.66 min was isoglycyrrhizin, and the peak with a retention time of 41.235 min was glycyrrhizic acid. The peak at 56.485 min was glycyrrhizin chalcone a, and the peak at 59.08 min was glycyrrhizin.

**Figure 8:**
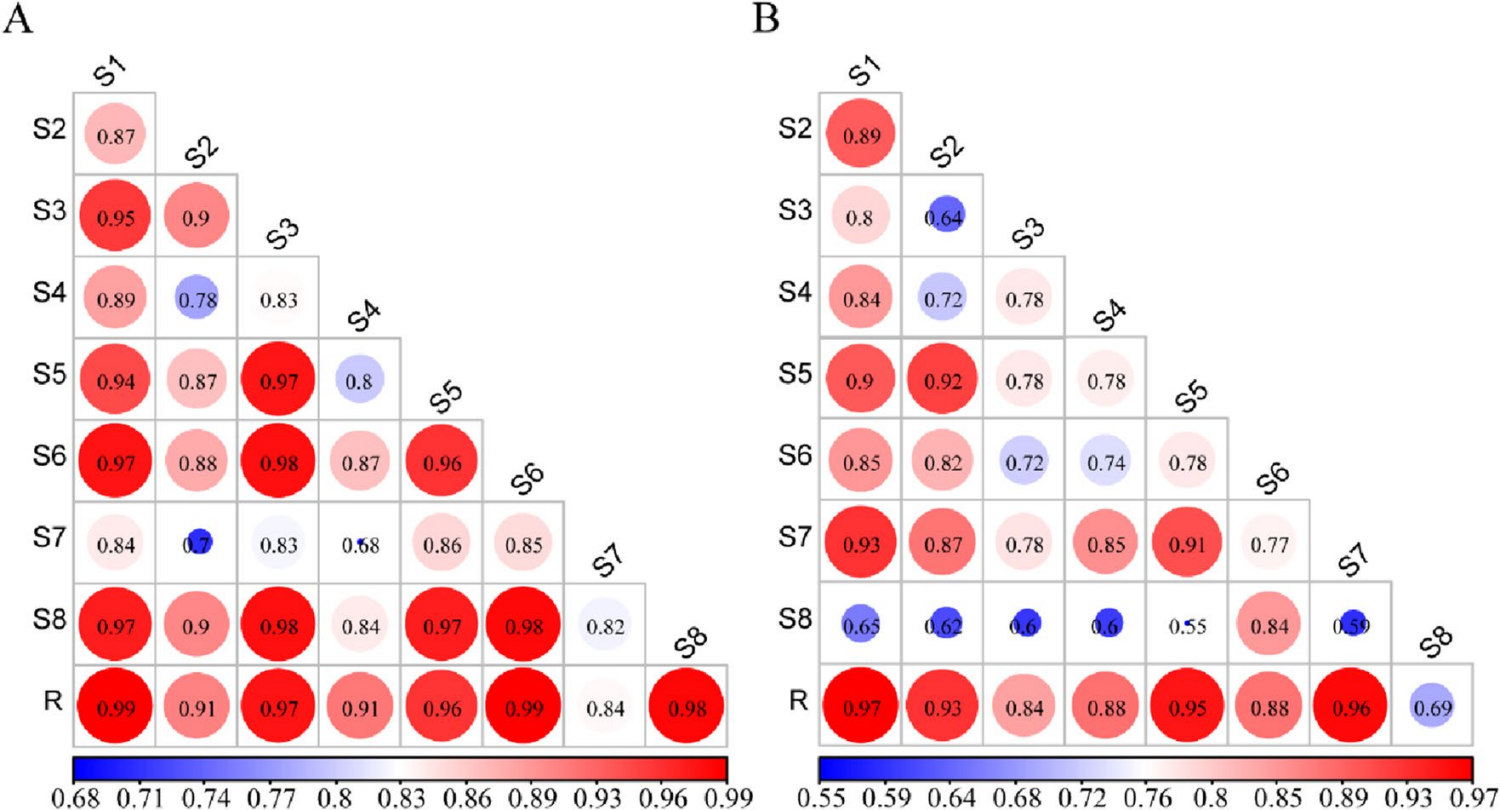
Comparison of the HPLC fingerprint similarity of licorice from the same provenance and different production areas (**A**-270 nm; **B**-365 nm).

Eight samples were compared at wavelengths of 270 and 365 nm to obtain Fig. 7D and E. The number and area of peaks in S7 and S8 were less than those in the other groups (Fig. 7D and E). The similarity evaluation is shown in Fig. 8. The similarity of the control fingerprints of *G. uralensis* from different production areas was 0.835–0.987 (270 nm) and 0.691–0.971 (365 nm), and the lowest similarity was observed for S7 (Baicheng, Jilin, 0.835) and S8 (Suide, Shaanxi, 0.691), respectively.

### Correlations of licorice yield and quality with climate and soil factors

**Figure 9:**
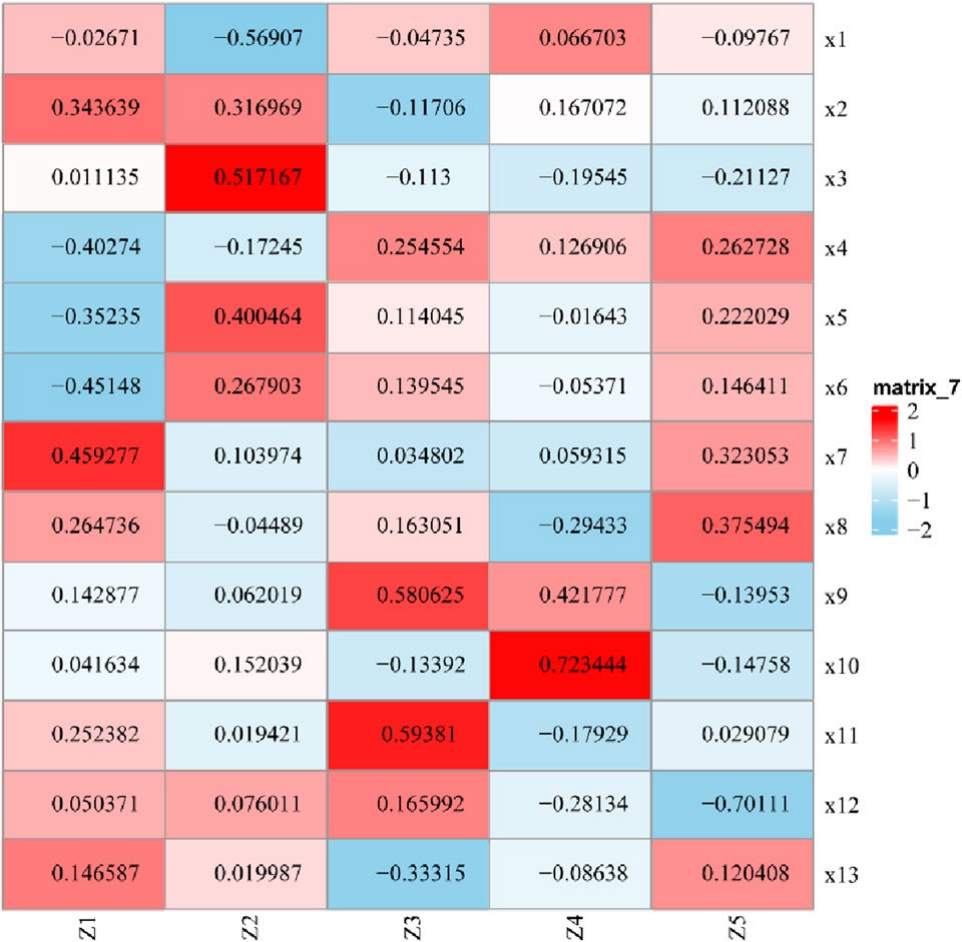
Load factor after rotation. The score formula of each factor is Zi = Σbjixj (i = 1, 2, …, 5; j = 1, 2, …, 13). bji is the coefficient in column i and row j of the factor score matrix, and xj is the standard value of the original variable.

Principal component analysis was conducted for 12 climate and soil factors and growth years (× 13) of all samples (n = 118). The results are shown in Table 2. The characteristic roots (Z1–Z5) with a cumulative contribution rate greater than 80% were selected (Fig. 9). Only a few variables in each factor had a large factor load (load coefficient). Larger values of the factor load indicate stronger correlations between the factor and variable and greater representativeness of the factor by the variable. If the factor load on several variables is large, the factor is composed of these variables. The variables with large factor loads in each factor were extracted, and then the factors were named according to the nature of the variables.

**Table 2:** Characteristic roots and contribution rate of each component.

Variable	Characteristic root	Contribution rate (%)	Cumulative contribution rate (%)
× 1	3.450191	0.2654	0.2654
× 2	2.854394	0.2196	0.485
× 3	1.574115	0.1211	0.6061
× 4	1.361012	0.1047	0.7107
× 5	1.17486	0.0904	0.8011
× 6	0.984552	0.0757	0.8769
× 7	0.811124	0.0624	0.9392
× 8	0.43306	0.0333	0.9726
× 9	0.184302	0.0142	0.9867
× 10	0.100472	0.0077	0.9945
× 11	0.04996	0.0038	0.9983
× 12	0.016399	0.0013	0.9996
× 13	0.005561	0.0004	1

× 1, altitude; × 2, longitude; × 3, latitude; × 4, annual average temperature; × 5, average temperature in July; × 6, highest temperature in the warmest month; × 7, annual precipitation; × 8, precipitation in the driest season; × 9, soil pH; × 10, soil available water grade; × 11, soil cation exchange capacity; × 12, soil organic carbon content; and × 13, growth years.

In characteristic root 1, × 6 and × 7 had larger load coefficients, which were the maximum temperature and average annual precipitation in the warmest month, so they were referred to as meteorological factors. × 1 and × 3 were the largest load factors in characteristic root 2, which were altitude and latitude, respectively; they were thus referred to as geographical factors. × 9 and × 11 had larger load coefficients on characteristic root 3, which were soil pH and soil cation exchange capacity, respectively; they were thus referred to as soil charge factors. × 10 was the largest load coefficient in characteristic root 4, which was the grade of soil available water and was referred to as the soil water factor. The largest load coefficient in characteristic root 5 was × 12, which was the content of soil organic carbon and was referred to as the soil nutrient factor. The load coefficients of soil moisture and nutrients (both above 0.7) were the largest. Three of the five characteristic roots were related to soil, indicating that soil conditions (especially water and nutrients) had the strongest effect on the yield and quality of cultivated licorice.

## Discussion

Many factors affect the distribution of plants, including humans, climate, and the soil environment. Since the Han Dynasty, the distribution of wild licorice has gradually expanded. In the Han Dynasty, wild licorice was mainly concentrated around Shaanxi. The distribution of wild licorice currently extends to Kashgar and Aksu in Xinjiang in northwestern China and Harbin and Mudanjiang in Heilongjiang Province in northeastern China. Three main factors drive these distributional patterns. The first is humans, including changes in people's activities. Since the Han Dynasty, the breadth of human activities gradually increased, and this has likely led to increases in the area of licorice cultivation. War can damage the growth environment of licorice, and damage and losses due to war have been mentioned in many ancient documents^27,28^. A large amount of domestic licorice was exported after the end of World War I because of excessive mining and excavation^29^ and this has led to the gradual depletion of wild licorice resources in some areas. The second factor is climate change. Resources can become increasingly exhausted when species are unable to adapt to changes in climate conditions^30,31^. For example, licorice was distributed in Jingzhou, Hubei Province in the Song Dynasty, but it is not present there today. The third factor is a change in soil conditions. Soil conditions have an important effect on changes in the distribution of plants^32,33^, such as changes in soil quality, soil moisture conditions, and nutrient elements. During this study, coal mines were excavated near the wild licorice fields in some areas, and this has resulted in a decline in the groundwater level. In addition, a reduction in local rainfall seriously affects the normal growth of licorice, and the density of local wild licorice has decreased annually.

Soil moisture factors have important effects on the growth and effective components of licorice. The results of this study show that the coefficient of the soil moisture factor was the largest. The growth of *G. uralensis* at the Jingtai experimental site was the highest, but the content of effective components of *G. uralensis* was low at this site, indicating that the environmental conditions in the Jingtai area are conducive to the growth of *G. uralensis* but not conducive to the accumulation of effective components. Although the soil texture, duration of the experiment, prior crop history, and diseases and insect pests were the same among all plots, the yield of the Jingtai site in Gansu Province was significantly higher than that of the other groups. A base watering pipe was located next to the test site. Although no direct irrigation was applied, some irrigation water was immersed in the test site, which might have affected the yield. A principal component analysis of environmental and soil factors confirmed that the soil water factor had an important effect on the yield and quality of licorice. The soil water content is known to have a key effect on the formation of plant yield and quality^34,35^, and plant photosynthesis is particularly sensitive to water^36–39^. Low water conditions affect leaf shape, stomatal conductance, and the ability of leaves to fix CO_2_, which affects plant growth^40–42^. Sufficient water is necessary for the accumulation of glycyrrhizic acid and glycyrrhizin^43^. Liu Changli et al. suggest that the yield and quality of cultivated Glycyrrhiza are relatively high when the relative water content of the soil is 50%^44^. The response of glycyrrhizic acid to changes in the soil water content is complex. Most studies have shown that the effect of the soil water content on glycyrrhizic acid first increases and then decreases, and the optimal range of the soil water content was 10–18%^45^.

The importance of soil moisture is second only to soil nutrients. This indicates that soil nutrient elements are important to the formation of licorice yield and quality. Sufficient soil carbon content is conducive to the growth of licorice and the accumulation of effective components^46,47^. Therefore, appropriate fertilization regimes will improve the yield and quality of licorice. For example, the application of a certain amount of biological carbon^48^ to the soil can improve the content of soil nitrogen, phosphorus, potassium, and total carbon^49^, alter soil pH^50^, promote increases in the abundance of beneficial fungi^51^, and Rhizobium nodulation, thereby enhancing crop yield^52^. Licorice mostly grows in desert areas, and biochar can improve the resistance of licorice to drought stress^53,54^. In addition to carbon, nitrogen, phosphorus, potassium, and medium trace elements can also increase the yield and quality of licorice. Fu et al.^55^ found that the content of effective components of *G. uralensis* was the highest when the nitrogen, P_2_O_5_, and K_2_O application rates were 238.81 kg hm^−2^, 119.40 kg hm^−2^, and 0 kg hm^−2^, respectively, in the Yanchi area of Ningxia. Ji et al.^56^ found that the recommended application of nitrogen, phosphorus, and potassium combined with the yield-increasing formula was 171 kg hm^−2^, 292.5 kg hm^−2^, and 49.5 kg hm^-2^, respectively, in an experimental site in Wuwei City, Gansu Province, which is consistent with the conclusions obtained by Zhang^57^ at the same experimental site. Zhang^58^ and Jin^59^ both showed that the combined application of nitrogen, phosphorus, and potassium can improve the yield and quality of licorice. After using exogenous calcium, the content of malondialdehyde in licorice was reduced, and the activities of superoxide dismutase, peroxidase, and catalase were increased, which enhanced the drought resistance of licorice and improved the yield and quality. Trace elements can increase the content of the effective components of licorice by promoting the activity of antioxidant enzymes, such as silicon, manganese, and molybdenum^60–63^.

In conclusion, the results of this study provide new insights into changes in licorice production areas; they also have implications for the selection of licorice production areas and future research and development of high-quality cultivation techniques.

## Conclusion

Wild licorice in China is mainly distributed in northern areas such as Gansu, Ningxia, and Inner Mongolia Provinces. The origin of wild licorice varies among periods. The origin of cultivated licorice in 59.26% of the cities that possess it was the same as that of wild licorice. The distribution of cultivated licorice was shifted to the northwest relative to that of wild licorice. Investigation of the yield and quality of cultivated licorice in China revealed substantial variation in the yield and quality of cultivated licorice in different production areas, and geographic variation was observed from west to east. The same batch of licorice seedlings was planted in 8 main licorice production areas in China. Differences in the yield, active ingredient content, and HPLC fingerprints were evaluated. The yield and active ingredient content of licorice in the Baicheng experimental site, Jilin Province were low; the growth of licorice in the Jingtai and Altay experimental sites was the highest, but the active ingredient content was low. The content of active components of licorice in Chifeng and Yuzhong experimental plots was higher, but the yield was lower. The similarity of the HPLC fingerprints of licorice from different production areas was 0.835–0.987 (270 nm) and 0.691–0.971 (365 nm), and the lowest similarity was 0.835 in Baicheng, Jilin Province, and 0.691 in Suide, Shaanxi Province. Principal component analysis of environmental and soil factors generated five characteristic roots with a cumulative contribution rate of 80%, three of which were related to soil and referred to as soil charge factor, soil water factor, and soil nutrient factor. The load coefficient of the water and nutrient factor was the largest, indicating that soil conditions had a substantial effect on the yield and quality of cultivated licorice. Changes in licorice production areas in different periods might be greatly affected by soil conditions, especially water and nutrients.

In sum, soil conditions, especially water and nutrients, might have a substantial effect on the observed changes in the licorice production area. Generally, the regulation of water and nutrients merits special attention when selecting areas for the production and cultivation of licorice.

## Materials and methods

All plant experiments were performed by relevant guidelines and regulations.

### Experimental materials

The experimental materials were 1-year-old licorice seedlings of the same source and the same specification. All seedlings were identified as *G. uralensis* Fisch. by professor Wang Wenquan of the Institute of Medicinal Plants, Chinese Academy of Medical Sciences.

### Experimental design

The China National Herbarium of Science and Technology (http://www.cvh.cn/), National Herbarium of Science and Technology (http://mnh.scu.edu.cn/), National Natural Science and Technology Resources Platform (http://168.160.153.204/Resource/), and relevant literature were used to determine the geographical distribution of licorice. Locations for sampling were located in the main areas of the distribution of licorice, which included Hohhot, Xinjiang, Jiuquan, Gansu, Lanzhou, Ningxia Wuzhong, Yulin, Shaanxi, Hangjin Banner, Chifeng, Inner Mongolia, Baicheng, and Jilin. Searches for licorice plants were based on this background information.

Three quadrats, which served as three replicates, were randomly assigned in each production area. The area of each quadrat was 9 m2. Nine plants in the quadrat were randomly selected for measurements of fresh weight and the content of glycyrrhizic acid and glycyrrhizin after excavation.

Specific survey locations are provided in Table S1. 

### Field experiment

In mid and late April, the same batch of annual licorice seedlings was transplanted into eight experimental sites in Altay, Xinjiang (ALT); Aksu, Xinjiang (AKS); Jinta County, Jiuquan, Gansu (JTA); Yuzhong County, Lanzhou, Gansu (LZ); Jingtai County, Baiyin, Gansu (JTG); Suide County, Yulin, Shaanxi (SD); Ongniute Banner, Chifeng, Inner Mongolia (CF); and Platform Town, Baicheng, Jilin (BC). The experiment was conducted in a randomized block design with three repetitions, and a total of 24 pilot plots. The community area was 25 m^2^, the isolation zone was 0.5 m, and the plant row spacing was 20 + 30 cm. The soil type of each experimental site was loam and the same cultivation and field management techniques were used across all sites. Before transplanting, no base fertilizer was applied to licorice at each test site, and plants were watered after they were transplanted. After that, only conventional field weeding was conducted, and no fertilizer or pesticides were applied.

### Sampling method

Sampling began in the middle of October of the year, and 10 plants were randomly excavated from each community to remove soil and other impurities.

### Indexes and measurements

#### Indexes

Root length, reed head diameter, fresh weight of single root, total flavonoids content, total saponins content, glycyrrhizin content, and glycyrrhizic acid content were measured, and the high-performance liquid chromatography (HPLC) fingerprint was taken. The longitude, latitude, and altitude of licorice were taken at the experimental site.

#### Measurements

##### Licorice yield index

The definition and method for determining the licorice yield index are provided in Table 3. The roots were excavated, the root shape index, root length, and root thickness were measured, and the fresh weight of the root was taken. Vernier calipers were used to measure the diameter of the reed head and the section diameter at 1 cm away from the reed head, and an electronic balance (accuracy 0.01 g) was used to measure the fresh weight of single roots.

**Table 3:** Definition of several variables used to determine the licorice yield index.

Variable	Unit	Definition
Root length	cm	Longest distance from the reed head to the root
Reed head diameter	mm	Root diameter 1 cm from the reed head
Root weight per plant	g	Weight of the root of the soil removed after excavation

##### Determination of the active components in licorice samples

After the licorice samples was dried, they were cut into sections, sampled by the quartering method, crushed, and sieved through a 60-mesh filter. The methods used to determine the content of total flavonoids, total saponins, glycyrrhizin, and glycyrrhizic acid, as well as the HPLC fingerprint of Glycyrrhiza are shown in Annex 1.

### Selection of climatic and soil factors

All the meteorological and soil index data used in this paper were from a database of spatial information on traditional Chinese medicine resources, including ecological factor data on the distribution of traditional Chinese medicine resources and data on climate and soil composition. The specific climatic and soil factors were as follows: altitude (× 1), longitude (× 2), latitude (× 3), annual average temperature (× 4), the average temperature in July (× 5), the maximum temperature in the warmest month (× 6), average annual precipitation (× 7), precipitation in the driest season (× 8), soil pH (× 9), soil effective water grade (× 10), soil cation exchange capacity (× 11), and soil organic carbon content (× 12) (Table 4).

**Table 4:** Climate and soil type data.

Variable	Unit
Meteorological factors	
Average temperature in July	°C × 10
Annual average temperature	°C × 10
Hottest month	°C × 10
Average annual precipitation	mm
Precipitation in the driest season	mm
Soil factor	
pH	–
Cation exchange capacity of soil	cmol/kg
Soil available water content grade	1
Soil organic carbon content	%

### Data analysis

Statistical analyses were performed in SAS 80, DPS 7.05, MS Excel 2016. R was used to make the graphs for the correlation analysis.

## Supplementary Information

Supplementary Information.

## Data Availability

The original contributions presented in the study are included in the article/supplementary material, further inquiries can be directed to the corresponding authors.
