# Supplementary material for: Study of the distribution of Glycyrrhiza uralensis production areas as well as the factors affecting yield and quality
Source: Sci Rep. 2023 Mar 29;13:5160. doi: 10.1038/s41598-023-31946-5 (PMC10060575; doi:10.1038/s41598-023-31946-5)
Supplement: Supplementary file 1 — Supplementary Information. [file 41598_2023_31946_MOESM1_ESM.pdf]

Supporting Information

Study of the Distribution of *Glycyrrhiza uralensis* Production Areas as well as the Factors Affecting Yield and Quality

Xinping Cui[1], Lin Lou[2], Yan Zhang[1*], Binbin Yan [1*]

1 State Key Laboratory and Breeding Base of Dao-di Herbs, Resource Center of Chinese Materia Medica China Academy of Chinese Medical Sciences, Beijing, 100700, China

2 School of Chinese Pharmacy, Beijing University of Chinese Medicine, Beijing 100102, China

*Correspondence

Yan Zhang E-mail: zhangyan8669@ 126. com

Binbin Yan E-mail: yb51598@126.com

	Place of Origin	Altitude/m	Longitude	Latitude
Xinjiang Uygur Autonomous Region	Wensu County, Aksu Prefecture	1008	79.96694	41.22222
	184 regiment of Tacheng and buxair County	548	86.515381	46.097138
	Changji City	482	85.29236	45.02306
	Fuyun County, Altay City	734	88.80617	46.38544
	Alar City	1014	81.1985	40.5527
	Karamay City	408	85.7072	46.8033
Gansu Province	Guazhou County, Jiuquan City	1160	95.7289	40.4769
	Yumen City	1480	96.9719	40.3631
	Gaotai County, Zhangye City	1480	99.3919	39.3558
	Changning Township, Wuwei City	1360	102.5569	38.4675
	Minqin County, Wuwei City	1340	102.6264	38.6257
	Shangshawo Town, Jingtai County, Baiyin City	1660	104.2464	37.415
	Huining Township, Yuzhong County, Lanzhou City	2328	104.4988	36.0272
	Yuzhong County, Lanzhou City	2415	104.84	36.09
	Hedong Township, Guazhou County, Jiuquan City	1365	96.7578	46.5269
	Xiaqinghe Township, Guazhou County, Jiuquan City	1349	98.52	39.7512
	Jinta County, Jiuquan City	1240	98.8829	40.1403
Ningxia Hui Autonomous Region	Hongsibao District, Wuzhong City	1157	106.0239	37.065
	Wuzhong Hongsibao Development Zone	1239	106.1441	37.4133
	Yuwang Town, Tongxin County, Wuzhong City	1540	106.3615	36.8008
	Gaoshawo Town, Yanchi County, Wuzhong City	1367.5	106.8506	37.9953

	Place of Origin	Altitude/m	Longitude	Latitude
	Wuzhong Hongsibao Development Zone	1169	106.1441	37.4133
	Xiamaguan Town, Tongxin County, Wuzhong City	1411	106.3817	36.8217
Shaanxi Province	Suide County, Yulin City	1054.1	110.561	37.819
Inner Mongolia Autonomous Region	Dengkou County, Linhe City, Bayannur	1030	106.9075	40.5767
	Rigalatu Town, Hangjin Banner	1017	107.8858	40.7714
	Duguitala Town, Hangjin Banner	1036	108.7479	41.2546
	Ulat Front Banner, Bayannur City	1037	108.814	40.6457
	Hangjin Banner, Ordos City	1068	108.937	41.0213
	Tumed Right Banner, Baotou City	990	110.6531	40.3183
	Heitutai Town, Fengzhen City	1481	113.7871	41.0512
	Niuyingzi County, Chifeng City	744	119.3458	43.0151
	Wengniute Banner, Chifeng City	705	119.5936	43.6222
	Naiman Banner, Tongliao City	380	120.741667	42.8241667
	Kezuohou banner, Tongliao City	190	122.160833	43.3727778
	Dalin Town, Horqin district, Tongliao City	150	122.762222	43.7630556
	Huhe Mudu Town, Hangjin Banner	1046	107.3118	40.5819
	Wulashan Town, Bayannur City	1037	108.814	40.6457
	Ulanhot Huhe Racecourse	122.08	122.4793	46.4052
Northeast China	Sihai forest farm, Taonan District, Baicheng City	160	122.4772	45.1247
	Platform Town, Baicheng City	190	122.7972	45.8753
	Shuguang village, platform Town, Baicheng City	166	123.3266	46.4552
	Shuishiying Manchu Town, angxi District, Qiqihar City	140	123.9775	47.0536
	Sihai forest farm, Taonan District, Baicheng City	160	122.4772	45.1247
	Platform Town, Baicheng City	165.5	123.3266	46.4552
	Nenjiang riverside, Tiefeng District, Qiqihar City	140	123.9558	47.4175
	Dawa town, Songyuan City	150	124.9444	45.3486

Table S1 List of study sites across the distribution of licorice

Province	City	Natural distribution area of wild licorice							Distribution of cultivated licorice producing areas
		Han Dynasty	The tang dynasty	Song dynasty	Yuan dynasty	The ming dynasty	Qing Dynasty	From the Republic of China to the present	
Shaanxi Province	Yulin	√	√	√	/	√	√	√	√
	Baoji	/	√	/	/	/	√	/	/
	Xianyang	/	/	√	/	/	/	/	/
	Chang'an	/	/	/	/	√	/	/	/
	Yan'an	/	/	/	/	/	√	√	/
Gansu Province	Wuwei	√	/	/	/	/	/	√	√
	Jiuquan	/	√	/	/	√	√	√	√
	Zhangye	/	√	/	/	/	√	√	√
	Lanzhou	/	/	√	/	/	√	/	√
	Baiyin	/	/	/	/	/	/	/	√
	Dingxi	/	√	√	/	√	√	√	/
	Tianshui	/	/	√	/	/	√	/	/
	Qingyang	/	/	√	/	√	/	√	/
	Pingliang	/	/	/	/	√	√	/	/
	Longnan	/	/	/	/	/	√	/	/
	Dunhuang	/	/	/	/	/	√	/	/
	Linxia	/	/	/	/	/	√	/	/
Ningxia Hui Autonomous Region	Lingwu	/	√	√	/	/	√	√	√
	Guyuan	/	/	√	/	/	√	/	/
	Wuzhong	/	/	/	/	/	√	√	√
	Zhongwei	/	/	/	/	/	√	/	/
	Shizuishan	/	/	/	/	/	/	√	/
Qinghai Province	Golmud	/	/	/	/	/	/	√	/
	Xining	/	/	/	/	/	√	/	/
Shanxi Province	Taiyuan	/	√	√	/	√	/	/	/
	Shuozhou	/	√	√	/	/	√	/	/
	Luliang	/	/	√	/	/	/	/	/
	Linfen	/	/	√	/	/	√	/	/
	Jincheng	/	/	√	/	/	/	/	/
	Jinzhong	/	/	/	/	/	√	/	/
	Changzhi	/	/	/	/	/	√	/	/
Inner Mongolia Autonomous Region	Ordos	/	√	/	/	/	/	√	√
	Hohhot	/	/	√	/	/	/	/	√
	Bayannur	/	/	/	/	/	/	/	√
	Chifeng	/	/	/	/	/	/	√	√
	Tongliao	/	/	/	/	/	/	√	√
	Alxa league	/	/	/	/	/	/	√	√
	Xilin Gol League	/	/	/	/	/	/	/	√
	Xing'an League	/	/	/	/	/	/	/	√
	Ulanqab	/	/	/	/	/	/	/	√
Three	Shenyang	/	/	/	/	/	√	√	/

northeastern provinces	Mudanjiang	/	/	/	/	/	√	/	/
	Haicheng	/	/	/	/	/	√	/	/
	Baicheng	/	/	/	/	/	/	√	√
	Harbin	/	/	/	/	/	/	/	√
	Chaoyang	/	/	/	/	/	/	√	/
	Jilin	/	/	/	/	/	/	√	/
	Changchun	/	/	/	/	/	/	√	/
	Zhaodong	/	/	/	/	/	/	√	/
	Daqing	/	/	/	/	/	/	√	/
	Qiqihar	/	/	/	/	/	/	√	/
	Songyuan	/	/	/	/	/	/	/	√
Xinjiang Uygur Autonomous Region	Kashgar	/	/	/	/	/	/	√	/
	Aksu	/	/	/	/	/	/	√	√
	Tacheng	/	/	/	/	/	/	/	√
	Changji	/	/	/	/	/	/	/	√
	Altay	/	/	/	/	/	/	√	√
	Alar	/	/	/	/	/	/	/	√
	Yili	/	/	/	/	/	/	√	√
	Karamay	/	/	/	/	/	/	/	√
Other provinces	LiaoCheng	/	√	/	/	/	/	/	/
	Qingdao	/	/	/	/	/	/	√	/
	Binzhou	/	/	/	/	/	/	√	/
	Tianjin	/	/	/	/	/	/	√	/
	Beijing	/	/	/	√	√	/	/	/
	Langfang	/	/	/	/	/	/	√	/
	Chengde	/	/	/	√	/	√	/	/
	Qinyang	/	/	/	√	/	/	/	/
	Jingzhou	/	/	√	/	/	/	/	/

Table S2 Cities with wild licorice and cities with cultivated licorice during different dynasties

Annex S1 Determination of the active components in licorice samples

1. Determination of total flavonoids

Experimental instruments and reagents: UV1700 Series UV-Vis Spectrophotometer (Shanghai Austrian Analytical Scientific Instrument Co., Ltd.); Ohaus cp214 Electronic Analytical Balance (Ohaus Instrument Co., Ltd.); Cpa225d 1 / 100000 Electronic Analytical Balance (Saidoris, Germany); Xl-06a Stainless Steel traditional Chinese Medicine Pulverizer (Guangzhou Xulang Machinery Co., Ltd.).

The reference substance glycyrrhizin (batch No. b20414) was purchased from Shanghai Yuanye Biotechnology Co., Ltd., and the purity was greater than 98%. Methanol, deionized water, and other reagents were analytically pure (Beijing Chemical Plant).

Preparation of the reference solution: Glycyrrhizin standard (0.900 g) was placed in a 10-ml volumetric flask; it was then dissolved in methanol, and a glycyrrhizin reference solution with a concentration of 0.0900 mg • ml^{-1} was prepared.

Preparation of the standard curve: Various volumes (0.5 ml, 0.75 ml, 1 ml, 1.5 ml, and 2.0 ml) of the above reference solution were placed into five 10-ml volumetric flasks. One ml of methanol was added, followed by 0.5 ml of 10% NaOH for color development; they were then placed at room temperature for 5 min and dissolved with methanol. The same procedure but without the reference solution was conducted as a blank control, and the absorbance value was measured at a wavelength of 334 nm. The linear regression equation with glycyrrhizin concentration C (mg/ml) as the abscissa and absorbance a as the ordinate was as follows: $y = 52.092x - 0.0042$ ($R^2 = 0.9999$, linear range 40 – 160 μg) (Figure S1).

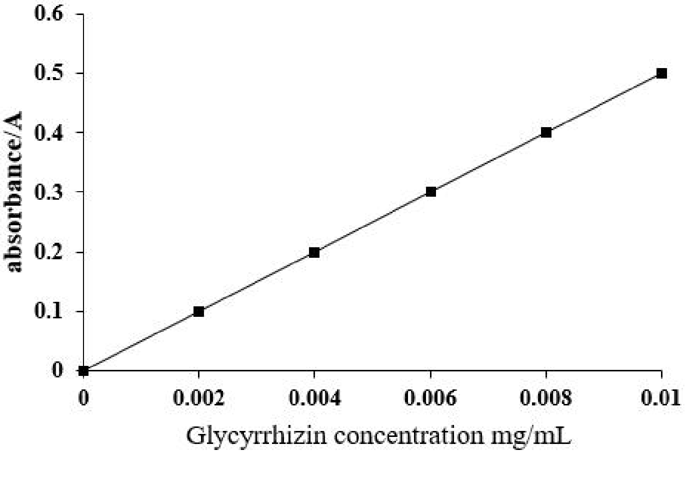

Figure S1 Standard curve of the total flavonoids of licorice

Determination of total saponins

Experimental instruments and reagents: UV1700 Series UV-Vis Spectrophotometer (Shanghai Aoxie Scientific Instrument Co., Ltd.); Semefi u3000 High-performance Liquid Chromatograph (USA); Ohaus cp214 Electronic Analytical Balance (Ohaus Instrument Co., Ltd.); Cpa225d 1 / 100000 Electronic Analytical Balance (Saidoris, Germany); and Xl-06a Stainless Steel Traditional Chinese Medicine Pulverizer (Guangzhou Xulang Machinery Co., Ltd.).

The reference glycyrrhizic acid (batch No. b20417) with a purity greater than 98%, was purchased from Shanghai Yuanye Biotechnology Co., Ltd. Methanol, deionized water, and other reagents were analytically pure (Beijing Chemical Plant).

Preparation of 5% vanillin acetic acid solution: Five g of vanillin was placed in a 100-ml brown volumetric flask; glacial acetic acid was then added and dissolved, and the volume was fixed to the scale.

Preparation of the reference solution: The glycyrrhizic acid reference solution (1.300 mg) was placed into a 10-ml volumetric flask; methanol was then added and dissolved.

Preparation of the standard curve: Various volumes (0.25 ml, 0.5 ml, 0.75 ml, 1 ml, 1.25 ml, 1.5 ml, 1.75 ml, and 2 ml) of the reference solution were placed in eight 10-ml volumetric flasks; after the methanol solution was volatilized, 0.2 ml of 5% vanillin glacial acetic acid solution was added, followed by 0.8 ml of perchloric acid. The solution was then shaken well, heated in a 55 ℃ water bath for 20 min, and cooled to room temperature. The volume was then fixed to the scale with glacial acetic acid and shaken well. The absorbance value was measured at 589 nm with the solvent without stock solution as the blank. The linear regression equation with mass (mg) of the reference substance as the abscissa and the absorbance as the ordinate was as follows: y = 14.872x - 0.0019 (R^2 = 0.9986, linear range 32.5–227.5 μg) (Figure S2).

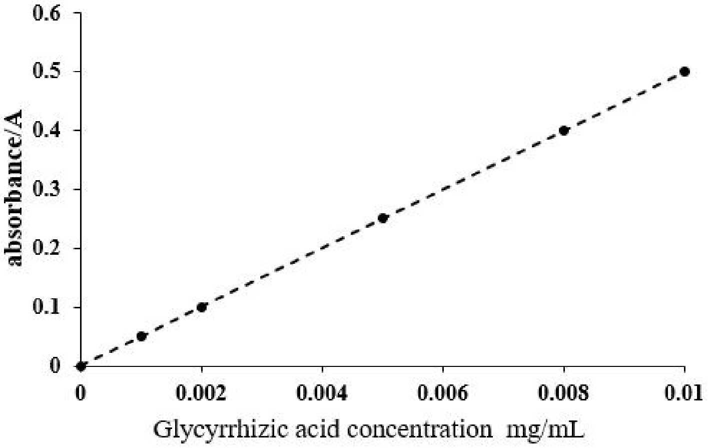

Figure S2 Standard curve of total glycyrrhizin

Determination of the glycyrrhizic acid and glycyrrhizin content

Test instrument and reagent: Semefi U3000 High-performance Liquid Chromatograph (USA); Ohaus cp214 Electronic Analytical Balance (Ohaus Instrument Co., Ltd.); Cpa225d 1 / 100000 Electronic Analytical Balance (Saidoris, Germany); Xl-06a Stainless Steel Traditional Chinese Medicine Pulverizer (Guangzhou Xulang Machinery Co., Ltd.); Kq500de Numerical Control Ultrasonic Cleaner; and zero point four five μM needle cylinder microporous membrane filter.

The standards of glycyrrhizic acid and glycyrrhizin were the same as those used for "total flavonoids" and "total saponins". Acetonitrile and phosphoric acid were chromatographically pure; ethanol was analytically pure, and Wahaha purified water was used.

Preparation of the reference solution: Glycyrrhizic acid (1.74 mg) and glycyrrhizin (1.26 mg) were placed into a 10-ml volumetric flask and dissolved in 70% ethanol, and the volume was fixed to the scale. The mobile phase was dissolved and diluted to the scale and shaken well. The control stock solution with a glycyrrhizic acid concentration of 0.174 mg · ml-1 and a glycyrrhizin concentration of 0.126 mg · ml-1 was obtained.

Preparation of the licorice test solution: Approximately 0.1 g of licorice sample powder (dried and crushed through a 60-mesh sieve) was placed in a conical flask with a stopper. Next, 50 ml of 70% ethanol was added; after the stopper was closed, the weight was taken. Ultrasonic treatment (power 250 W, frequency 40 KHz) was conducted for 30 min; after cooling, the weight was taken again, and the lost weight was made up with 70% ethanol. The solution was then shaken well and filtered, and the continuous filtrate was withdrawn.

Chromatographic conditions: Following the method described in Pharmacopoeia 2020, a Diancmsi (R) Diamond C18 chromatographic column (250 mm × 4.6 mm, 5 μm) was used. Acetonitrile-0.05% phosphoric acid water

was used as the mobile phase, the flow rate was 1 ml · min-1, and the column temperature was 30 ℃; gradient elution was conducted according to the elution procedure in Table S3. The detection wavelength was 237 nm, and the injection volume was 10 μl. The number of theoretical trays should not be less than 5,000 according to the glycyrrhizin peak.

Time (min)	Acetonitrile (%)	0.05% phosphoric acid water (%)
0–8	19	81
8–35	19→50	81→50
35–36	50→100	50→0
36–40	100→19	0→81
40–50	19	81

Table S3 Mobile phase gradient conditions

Investigation of the linear range: First, 0.5% of glycyrrhizin and glycyrrhizic acid mixed reference solution (1 μL, 5 μL, 10 μL, and 20 μL) was injected, and the peak area value was determined. The standard curve was drawn with injection volume (μg) as the abscissa and peak area as the ordinate. The regression equation of glycyrrhizin and glycyrrhizic acid is shown in Table S4.

Reference substance	Regression equation	R2	Linear range/μg
Liquiritin	y = 26.072x + 0.3345	0.9995	0.063~2.52
Glycyrrhizic acid	y = 10.637x + 0.1848	1	0.087~3.48

Table S4 Standard curve of glycyrrhizin and glycyrrhizin

Precision experiment: The test solution was absorbed, samples were injected 6 times, and the chromatographic peak area was determined. The RSD of the content of glycyrrhizin and glycyrrhizic acid was 1.81% and 1.17%, respectively, indicating that the precision of the instrument was high.

Stability test: The same sample solution was injected at 0 h, 3 h, 6 h, 12 h, 24 h, and 48 h. The stability of the sample solution was high within 48 h. The RSD of glycyrrhizin and glycyrrhizic acid was 1.67% and 2.54%, respectively, indicating that the sample solution was stable within 48 h.

Repeatability experiment: The licorice sample powder was weighed, and 6 sample solutions were prepared. According to the above method, the relative standard deviation values of glycyrrhizin and glycyrrhizic acid were 0.6% and 1.56%, respectively, indicating that the repeatability of the method was adequate.

4. HPLC fingerprint

Test instruments and reagents: A Semefi u3000 High-performance Liquid Chromatograph and a Diamonsi (R) Diamond C18 column (250 mm) × 4.6 mm, 5 μm were used. The mobile phase was acetonitrile (a) - 0.1% phosphoric acid water (b). The control samples of apigenin, glycyrrhizin, apigenin isoglycyrrhizin, isoglycyrrhizin, isoglycyrrhizin, glycyrrhizic acid, glycyrrhizin chalcone A, and Glycyrrhiza glabra were obtained from Shanghai Yuanye Biotechnology Co., Ltd. and the others were the same as the determination of glycyrrhizin and glycyrrhizic acid.

The elution procedure was as follows: 0 min, 15% a; 15 min, 25% A; 40 min, 38% A; 52 min, 50% A; 68 min, 74% A; and 80 min, 80% A. The flow rate was 0.8 ml · min-1, the column temperature was 30 ℃, the detection wavelengths were 270 and 365 nm, and the injection volume was 10 μL.

Preparation of the test article: The procedure was the same as that for glycyrrhizin and glycyrrhizic acid.

Configuration of the standard product: Appropriate amounts of celerose glycyrrhizin, glycyrrhizin, celerose isoglycyrrhizin, isoglycyrrhizin, glycyrrhizin, isoglycyrrhizin, glycyrrhizic acid, glycyrrhizin chalcone A, and glycyrrhizin were used to prepare the mixed standard; it was then dissolved with 70% ethanol and stored at 4 ℃ in a refrigerator.
